# Determinants of use of mobile phones for sexually transmitted infections (STIs) education and prevention among adolescents and young adult population in Ghana: implications of public health policy and interventions design

**DOI:** 10.1186/s12978-019-0763-0

**Published:** 2019-08-09

**Authors:** Robert Kaba Alhassan, Abdulai Abdul-Fatawu, Belinda Adzimah-Yeboah, Worlali Nyaledzigbor, Samuel Agana, Prudence Portia Mwini-Nyaledzigbor

**Affiliations:** 1 Department of Public Health Nursing, School of Nursing and Midwifery University of Health and Allied Sciences, Ho. PMB 31, Volta Region Ho, Ghana; 2 Former student, School of Nursing and Midwifery, University of Ghana Legon, Accra, Ghana; 3 Central University, Miotso Campus, Greater Accra Region Accra, Ghana; 4 ICT Directorate, University of Health and Allied Sciences, Ho, Ghana

**Keywords:** Education, Ghana, téléphones portables, population, prévention, santé publique, infections sexuellement transmissibles, adolescents, étudiants de premier cycle, jeunes adultes, Education, Ghana, Mobile phones, Population, Prevention, Public health, Sexually transmitted infections, Adolescents, Undergraduate, Young adult

## Abstract

**Background:**

Sexually Transmitted Infections (STIs) are a major public health challenge globally especially among adolescents and young adults in lower-middle-income countries (LMICs) in Africa including Ghana. In light of this, mobile phone innovations are advocated to enhance public health education and prevention of STIs in developing health systems.

**Objective:**

This study assessed mobile phone usage among adolescents and young adult populations pursuing tertiary education and their use of these technologies in the education and prevention of STIs.

**Method:**

This was a cross-sectional analytical study among 250 adolescents and young adults aged 18–24 at Ghana’s premier and biggest public University. The study was however conducted in only one public university in the Greater Accra region  which potentially poses generalizability challenges due to socio-cultural and economic differences in other regions of the country. Data was collected using structured questionnaire and data analysis done with STATA (version 12.0). Univariate probit regression (VCE, Robust) analysis was used to determine factors associated with adolescents and young adult population’s usage of mobile phones in the education and prevention of STIs.

**Results:**

Out of the 250 adolescents and young adults interviewed, 99% owned mobile phones. Out of this number, 58% them were smartphone users. Also, it was found that male young adults (Coef. = 1.11, *p* = 0.000) and young adults who owned a smartphone (Coef. = 0.46, *p* = 0.013) were more likely to use mobile phones for education and prevention of STIs.

**Conclusion:**

Mobile phone penetration among young adults is nearly 100% in line with the national trend. Additionally, these young adults largely believe in the use of mobile phone programmes for STIs education and prevention. Moreover, respondents were found to be more comfortable using mobile applications than traditional text messaging or phone calls in STIs education and prevention. Future mobile phone programmes for STIs education and prevention should consider innovating customized mobile applications to promote acceptability by the youth and enhance sustainability of such interventions on STIs in Ghana. Even though this study was conducted in only one public university in Ghana, the findings are nonetheless informative and future researchers could consider using a larger sample size across private and public universities in other regions of the country.

**Electronic supplementary material:**

The online version of this article (10.1186/s12978-019-0763-0) contains supplementary material, which is available to authorized users.

## Abstrait

**Contexte:**

Les infections sexuellement transmissibles (IST) constituent un problème de santé publique majeur dans le monde, en particulier chez les adolescents et les jeunes adultes des pays à revenu intermédiaire de la tranche inférieure des pays à revenu intermédiaire en Afrique, y compris le Ghana. Dans ce contexte, les innovations en matière de téléphonie mobile sont préconisées pour améliorer l'éducation en matière de santé publique et la prévention des IST dans les systèmes de santé en développement.

**Objectif:**

Cette étude a évalué l’utilisation du téléphone mobile chez les adolescents et les jeunes adultes poursuivant des études supérieures et l’utilisation de ces technologies dans le cadre de l’éducation et de la prévention des IST.

**Méthode:**

Il s’agissait d’une étude analytique transversale menée auprès de 250 adolescents et jeunes adultes âgés de 18 à 24 ans de la première et plus grande université publique du Ghana. L’étude a été menée dans une seule université publique, ce qui pose potentiellement des problèmes de généralisabilité en raison des différences socioculturelles et économiques entre les différentes régions du pays. Les données ont été collectées à l’aide d’un questionnaire structuré et leur analyse a été réalisée avec STATA (version 12.0). Une analyse de régression probit univariée (VCE, Robust) a été utilisée pour déterminer les facteurs associés à l’utilisation du téléphone portable par les adolescents et les jeunes adultes dans l’éducation et la prévention des IST.

**Résultats:**

Sur les 250 adolescents et jeunes adultes interrogés, 99% possédaient un téléphone mobile. Sur ce nombre, 58% étaient des utilisateurs de smartphone. En outre, il a été constaté que les jeunes adultes de sexe masculin (Coef. = 1,11, *p* = 0,000) et les jeunes adultes possédant un smartphone (Coef. = 0,46, p = 0,013) étaient plus susceptibles d’utiliser les téléphones mobiles pour l’éducation et la prévention des IST.

**Conclusion:**

Le taux de pénétration du téléphone portable chez les jeunes adultes est. presque 100% conforme à la tendance nationale. De plus, ces jeunes adultes croient largement en l’utilisation de programmes de téléphonie mobile pour l’éducation et la prévention des IST. De plus, les répondants se sont montrés plus à l’aise avec les applications mobiles que la messagerie texte ou les appels téléphoniques traditionnels pour l’éducation et la prévention des IST. Les futurs programmes de téléphonie mobile destinés à l’éducation et à la prévention des IST devraient envisager d’innover dans les applications mobiles personnalisées pour promouvoir l’acceptabilité par les jeunes et améliorer la durabilité de telles interventions sur les IST au Ghana. Bien que cette étude ait été menée dans une seule université publique du Ghana, les conclusions sont néanmoins informatives et les futurs chercheurs pourraient envisager d’utiliser un échantillon plus grand parmi les universités privées et publiques des autres régions du pays.

## Plain English summary

Sexually Transmitted Infections (STIs) are a major public health challenge globally especially among adolescents and young adults in low- and middle-income countries in Africa. In light of this, mobile phones innovations are being encouraged to help provide the needed education and prevention of STIs in developing countries such as Ghana. This study was conducted to assess mobile phone usage among adolescents and young adult population and their use of mobile phones to educate and prevent STIs. The study was conducted among 250 adolescents and young adults aged 18–24 at the University Ghana, Legon. At the end of the study it was found that out of the 250 adolescents and young adults interviewed, 99% owned mobile phones and 58% of these participants owned and used smartphones. It was found that being a male and owning a smartphone associated positively with adolescents and young adults’ probability of using mobile phones to educate themselves and others on prevention of STIs. It is recommended that future mobile phone programmes for STIs education and prevention should consider innovating customized mobile applications to promote acceptability by the youth who are worst affected by STIs in Ghana.

## Résumé en français simplifié

Les infections sexuellement transmissibles (IST) constituent un problème de santé publique majeur dans le monde, en particulier chez les adolescents et les jeunes adultes dans les pays à revenu faible et intermédiaire en Afrique. Dans ce contexte, les innovations en matière de téléphonie mobile sont encouragées à contribuer à l’éducation et à la prévention nécessaires des IST dans les pays en développement, tels que le Ghana. Cette étude visait à évaluer l’utilisation du téléphone mobile chez les adolescents et les jeunes adultes ainsi que leur utilisation du téléphone mobile pour éduquer et prévenir les IST. L’étude a été menée auprès de 250 adolescents et jeunes adultes âgés de 18 à 24 ans de l’Université du Ghana à Legon. À la fin de l’étude, il a été constaté que sur les 250 adolescents et jeunes adultes interrogés, 99% possédaient un téléphone mobile et 58% d’entre eux possédaient et utilisaient un smartphone. Il a été constaté que le fait d’être un homme et de posséder un smartphone était associé positivement aux chances des adolescents et des jeunes adultes d’utiliser leurs téléphones portables pour se renseigner et informer les autres sur la prévention des IST. Il est. recommandé que les futurs programmes de téléphonie mobile destinés à l’éducation et à la prévention des IST envisagent d’innover dans les applications mobiles personnalisées afin de promouvoir l’acceptabilité par les jeunes les plus touchés par les IST au Ghana.

## Background

Sexually Transmitted Infections (STIs) are a major public health challenge globally. STIs such as HIV/AIDS, gonorrhea, syphilis, herpes simplex virus, human papillomavirus, chlamydia, and vaginitis have been a major source of public health concern, particulalry in LMICs in Africa. Despite the active and passive surveillance activities and other interventions aimed at accelerating case finding, education, and treatment of cases, STIs remain a burden to many countries [1] including Ghana [2].

Adolescents and young adults in developing countries have been the worst affected by STIs over the years [3]. This is worrying because young adults in developing nations account for 85% of the global young population [4]. According to a WHO report in 2015, the population of adolescents (10–19 years) in Africa stood at 224 million and this number is expected to double by the year 2050. Burden of STIs including HIV/AIDS in sub-Sharan Africa is worse since the sub-region has the highest percentage of STIs [5]. Despite sub-Saharan Africa being the region with the highest percentage of STIs among young adults, only 13% and 9% of adolescent boys and girls respectively got tested for STIs in the year 2016 [6]. Additionally, as per the 2014 Ghana Demographic and Health Survey (GDHS) report, knowledge levels among adolescents in Ghana on STIs remain low [7]. Moreover, one out of every eight girls aged 15–24 years have reported symptoms of STIs [7]. This represents the highest rate among all age groups thus predisposing adolescents and young adult population to a disproportionate risk of STIs.

In Africa, HIV/AIDS is a leading cause of death only second to lower respiratory tract infections [3]. Burden of STIs, particularly HIV/AIDS, in Ghana is equally alarming. According to the National AIDS Control Programmes (NACP) in Ghana [cited in 3], HIV prevalence in Ghana in 2017 was 1.7 population and 2.1 among antenatal care (ANC) attendants. An estimated 310,000 people were living with HIV/AIDS in 2017 out of which 28,000 (9%) were children between 0 and 14 years. Additionally, a total of 19,000 new cases of HIV were recorded in 2017 alone with 12,000 deaths and 18% of these deaths occurred among children under 14 years [2].

As part of efforts to address the epidemic of HIV/AIDS and STIs, in Ghana a National HIV/AIDS policy was formulated in 2013 with key intervention strategies and mechanisms for HIV and STIs prevention in Ghana [8]. The key strategies are: 1) creation of technical working groups on key populations, anti-retroviral therapy, research, monitoring and evaluation, communication and an expanded technical working group; 2) formation of task teams, such as gender and HIV, stigma reduction, prevention of mother-to-child transmission (PMTCT), decentralised response and World AIDS Day planning committees; 3) organization of the partnership forum; and 4) routine technical review meetings with implementing partners and stakeholders [8]. Unfortunately, full execution of these policy strategies has been impeded by limited resources over years.

In addition, although efficacious biomedical interventions in the treatment and prevention of STIs have been made available, these measures are not sufficiently effective in practice because biomedical interventions are not in themselves a solution to the problem but depend on behavioural and structural intervention components for their effectiveness [9]. In an attempt to provide comprehensive care for STIs, mobile phones have been used to provide the behavioural and structural intervention component needed for the care, education, and prevention of STIs [10–13].

In light of this, the WHO has demonstrated commitment in the use of mobile technologies such as eHealth and mHealth innovations to promote cost effective public health education on STIs including HIV/AIDS [14]. Use of Information and Communication Technology (ICT) innovations in health promotion activities has the potential to reduce healthcare costs to families and improve equitable access to quality STIs services. Moreover, ICT innovations have been proven to promote efficient linkage of health systems with social protection programmes, and increase accountability and sustainability in the health service delivery value chain [14].

The rapid proliferation of mobile phones in developing countries has created new channels of communication for the dissemination of health information to a greater number of people at a minimal cost [15, 16]. The increasingly sophisticated nature of these mobile phones provides a promising platform for adolescents and young adults to actively engage with health workers for reproductive health services. These mobile technologies have the potential for STIs prevention among the current digital generation of young adults [13]. This potential is even greater among adolescent and young adults with the rapid adoption of mobile phone technologies.

Previous studies conducted elsewhere and Africa have shown that mobile phones have been used in STI prevention and care activities to reduce high-risk behaviours, increase retention in care, promote prevention messages, improving result notification, medication adherence and clinic attendance [13, 17–19].

Similar mobile-Health (*mHealth*) projects have been implemented in the past  for the prevention and education of STIs among adolescents and young adults in Ghana. However, these projects were implemented without taking into account the interest, views, and consideration of the end users to avert acceptability and sustainability challenges largely because majority of eHealth and mobile-based innovations are donor funded and once the donors pull out such projects suffered financial and technical know-how constraints [20]. Moreover, there are limited studies done regarding adolescents and young adults’ interest in mobile phone-based programmes towards STIs prevention. It is therefore imperative that before scarce resources are invested for mobile phone-based services for STIs prevention, one needs to determine whether adolescent and young adults would be interested in using the services and which mobile phone technology they would prefer for STIs education.

This study sought to assess mobile phone ownership and the frequency of use among young adults aged 18–24 years at the University of Ghana, Legon; explore adolescents and young adult population usage of mobile phones for STIs education. Also, the study sought to establish the mobile phone technologies young adults prefer to use for STIs related programmes. Finally, the study explored factors associated with adolescents and young adults use of mobile phones for STIs education and prevention.

## Methods

### Study design, setting and population

The study was a cross-sectional analytical study conducted among adolescents and young population at the University of Ghana, Legon campus in July 2016. The University of Ghana, established in 1948, is the premier and largest university in Ghana and as at 2018 had 32, 918 population of undergraduate students offering Bachelors and sub-degree programmes while postgraduate students’ population was 4820 [21]. Postgraduate students were excluded from this because they are more likely to exceed 24 years of age than the undergraduate group category of students. The university operates the collegiate system comprising of the colleges of Basic and Applied Sciences, Education, Health Sciences, and Humanities [21]. The study population is not necessarily representative of the larger Ghanaian population due to socio-cultural and economic variations in the larger adolescent and young adult population in Ghana. However, since the study setting is the largest public university in Ghana with diverse caliber of international and local students across all regions of the country the composition still gives a fair reflection of the national situation.

### Sample size determination and participants recruitment

Using Krejcie and Morgan [22] formula for determining sample size based on known populations at 95% confidence level, 350 earmarked sample size was determined based on the population of undergraduate adolescents and young adults age groups (*N* = 32,059). Subsequently, respondents were recruited through a non-probability sampling technique (convenience), acknowledging the limitation of possible self-selection where adolescents and young adults not averse to mobile phone usage might have volunteered most to participate in the study. Adopting probability sampling technique such as simple random could have reduced this potential bias but given the busy academic schedules of study participants at the time of conducting this study, the convenience sampling approach (which is also scientific) was deemed more appropriate to attain reasonable sample size for the study. Moreover, relevant statistical analysis strategies were adopted to mitigate the potential effect of the chosen sampling method.

### Data collection instruments

Structured questionnaires were used for the data collection. Adolescents and young adult population were asked to complete these questionnaires anonymously to maintain privacy and confidentiality of respondents. Response to the tools was self-reported by the respondents in English since they were all literate in the English Language. The structured questionnaires were piloted and peer reviewed for validation. Piloting of the tools was done in a comparable tertiary institution outside the study setting to avoid possibly sensitizing the respondents to the pattern of the questions. Outcome of the piloting was a review of some ambiguous questions and correction of typographical errors. The questionnaire had a total of 22 questions grouped into three sections: Section A: Socio-demographic features of respondents; Section B: Beliefs, interests and usage of mobile phones for STIs education and prevention, and Section C: Factors associated with respondents’ usage of mobile phones for STIs education, prevention and control. The questionnaires varied from dichotomous responses of “Yes” or “No” to Likert scale items. Eight (8) questions on frequency and ease of use of mobile phones for promoting HIV/AIDS education were framed on a five-point Likert scale from 1 “very frequently” to 5 “never”. Please see details of questionnaire in the attached Additional file 1. Scale reliability of the Likert scale items was checked and found to be 0.72 which is acceptable according to Bleda and Tobias [23].

### Operational definition of terms

Use of mobile phone for STIs education or prevention activities: In this context meant, students who self-reported whether or not they use their mobile phones for the purpose of accessing educational materials on STIs and their prevention. This variable had a binary response outcome “1 = Yes” or “0 = No”. The limitation is that there was no independent verification of the study participants’ responses.

Frequency of use of mobile phone on daily basis: In this context meant the self-reported frequency (in terms of number of hours) of use of the mobile phone in a day. This variable was captured in categorical outcome (i.e. approximate intervals of usage in hours). Likewise, there was no independent means of verifying these responses.

### Ethical consideration

Ethical clearance was obtained from the institutional review board of the College of Basic and Applied Sciences of the University of Ghana, Legon. Informed consent from  participants of this study was written consent since all respondents were literates who could read and write.

### Data collection and analysis

Out of the 350 administered questionnaires, a total of 275 questionnaires were retrieved. Out of the 275 retrieved questionnaires, 250 of them had complete information with signed informed consent forms. Thus, the sample size used for the final analysis was 250, representing a return rate of 71%. All administered questionnaires were checked for completeness, coded (for anonymity) and capture into Microsoft Excel Spreadsheet before exporting to STATA statistical software (version 12.0) for final analysis. Descriptive analysis was  performed to describe background information of the respondents.

Chi-square test was performed to ascertain if there is any statistically significant association between the independent variables of interest which were later included in the univariate probit analysis. The univariate probit regression was conducted to ascertain predictors of respondents’ likelihood of using mobile phones for STIs education prevention or otherwise. Thus, main outcome variable of interest was the use of mobile phone for STIs education or prevention activities (a binary response “1 = Yes” or “0 = No”).

The key independent variables of interest were the gender of respondents (“1=Male” or “0=Female”), and type of mobile phone owned/used by respondents (“1 = Smart phone” or “0 = GSM phone”). Other variables controlled in the regression model were: age, educational level, programme of study, ownership of a mobile phone, and frequency of use of mobile phone on daily basis. Multicollinearity diagnostics was done on the all explanatory variables and the mean Variance Inflation Factor (VIF) was 1.04. Moreover, none of the explanatory variables recorded VIF up to 5 or 10, necessary for exclusion from the regression model [23].

## Results

Out of the 275 retrieved questionnaires, 250 were completely filled by respondents and informed consent forms duly signed. Out of the 250 respondents, 149 (59.6%) were males and in their fourth year of Bachelor studies, 57 (22.8%). The most represented ethnic affiliation was Akan, 117 (46.8%); while 248 (99.2%) of the respondents owned mobile phones at the time of this study (See Table 1).

**Table 1:** Association between gender and other characteristics of respondents (*n* = 250)

Characteristic	Female (n=101) f (%*)	Male (n=149) f (%*)	Total (n=250) f (%*)	*p*-value
Age				0.412
18–19	15 (14.9)	14 (9.4)	29 (11.6)	
20–21	16 (15.8)	39 (26.2)	55 (22.0)	
22–23	40 (39.6)	50 (33.6)	90 (36.0)	
24	30 (29.7)	46 (30.9)	76 (30.4)	
^a^Study Level			.	0.346
All years of Diploma studies	21 (20.8)	25 (16.8)	46 (18.4)	
1st Year of Bachelor Studies	16 (15.8)	28 (18.8)	44 (17.6)	
2nd Year of Bachelor Studies	25 (24.8)	29 (19.5)	54 (21.6)	
3rd Year of Bachelor Studies	20 (19.8)	29 (19.5)	49 (19.6)	
4th Year of Bachelor Studies	19 (18.8)	38 (25.5)	57 (22.8)	
Ethnic Affiliation				0.206
Akan	51 (50.4)	66 (44.3)	117 (46.8)	
Ga-Adangbe	12 (11.9)	26 (17.4)	38 (15.2)	
Ewe	13 (12.9)	26 (17.4)	39 (15.6)	
Dagomba	12 (11.9)	12 (8.1)	24 (9.6)	
Frafra	8 (7.9)	5 (3.4)	13 (5.2)	
Others	5 (5.0)	14 (9.4)	19 (7.6)	
Type of phone owned				0.016^†^
Smartphone	57 (56.4)	87 (58.4)	149 (57.6)	
Feature phone	44 (43.6)	62 (41.6)	101 (42.4)	
Brand of phone owned				0.006^†^
Nokia without android features	15 (14.9)	26 (14.2)	41 (16.4)	
Windows phone with android features	18 (17.8)	30 (24.4)	48 (19.2)	
Android phones of other brands	19 (18.8)	43 (34.6)	62 (24.8)	
iPhone	16 (15.8)	22 (15.7)	38 (15.2)	
Blackberry with android features	18 (17.8)	9 (11.0)	27 (10.8)	
Others phone brands without android features	15 (14.9)	19 (12.8)	34 (13.6)	
^**^Frequency of use of phone				0.011^†^
Every 2 h or less	44 (43.6)	62 (41.6)	106 (42.4)	
Every 3-4 h	16 (15.8)	35 (23.5)	51 (20.4)	
Every 5-6 h	10 (9.9)	23 (15.4)	33 (13.2)	
Every 7-8 h	19 (18.8)	16 (10.7)	35 (14.0)	
9 h or more	12 (11.9)	13 (8.7)	25 (10.0)	
Study Department				0.092
Business admin	27 (26.7)	52 (34.9)	79 (31.6)	
Health sciences	36 (35.6)	47 (31.5)	83 (33.2)	
Arts	12 (11.9)	16 (10.7)	28 (11.2)	
Science & technology	5 (3.4)	5 (5.0)	10 (4.0)	
Others departments	21 (20.8)	29 (9.5)	50 (20.0)	

Source: Field Data (2016)

Legend: ^†^*p* < 0.05; N (Number of valid responses); ^a^Study Level (In Ghana this represents the 4 years of University studies required to complete a first degree or Bachelor degree; Diploma studies are relatively shorter usually 1 or 2 years ending up in the award of a Diploma, which is lower than a Bachelor Degree); *All percentages have been rounded-up to the nearest decimal point and percentages in column 1 computed using the number of valid responses for females (n=101) as the denominator; percentages in column 2 were computed using the number of valid responses for males (n=149) as the denominator; percentages in column 3 were computed using the total number of valid responses for females and males (n=250) as the denominator; **Frequency of use of phone includes use of phone on daily basis for health education and other daily activities of living

### Adolescents and young adult population’s perspectives on use of mobile phones for STIs education and prevention

Respondents who believed adolescents and young adults would be interested in using mobile phones for STIs education and prevention were categorized as being interested as against those not interested. This variable produced two dichotomous responses on respondents’ beliefs on adolescents and young adults’ interest in using mobile phones for STIs prevention. Seventy percent (70%) of the respondents indicated adolescents and young adults would be interested while 30% did not.

Fifty (20%) of the respondents indicated text messaging as the appropriate mobile phone function for STIs education and prevention; 101 (40.1%) indicated mobile applications; 41 (16.4%) said phone call; 43 (17.2%) preferred mobile web, and 15 (6%) said other mobile phone functions were the most appropriate means for STIs education.

### Frequency of using the mobile phone functions

Frequency with which respondents use the various functions of mobile phones was also assessed. Phone call was the most frequently used mobile phone function as 201 (80.4%) of the respondents said they make phone calls more frequently; 47 (18.8%) said they occasionally make a phone call and 2 (0.8%) respondents claimed they never made a phone call (perhaps these respondents did not honestly answer this question since there was no independent means of validating these responses, and the researchers acknowledge this limitation of the study).

Mobile phone applications were the next most frequently used mobile phone function where 162 (64.8%) said they frequently use this mobile phone function; 84 (33.6%) occasionally use mobile applications while 4 (1.6%) claimed they never used a mobile phone application. One hundred and fifty-five (62%) respondents indicated they frequently use mobile web; 89 (35.6%) occasionally use mobile web, and 6 (2.4%) respondents said they never used mobile web.

Text messaging was the least frequently used mobile phone function with 116 (46.4%) frequently engaged in text messaging; 133 (53.2%) occasionally text while 1 (0.4%) respondent indicated she does not use text messaging. The more occasional use of text messaging as a means of communication among the respondents elucidates the growing penetration of smartphones among the younger population with applications and web functionalities.

### Factors associated with respondents’ use of mobile phones for STIs education and prevention

Univariate Probit regression (VCE Robust) was conducted to ascertain the predictors of young adults’ use of mobile phone for the purposes of STIs education and prevention activities. The results of the regression analysis showed that the predictive probability of using mobile phone for STIs education and prevention is high among male adolescents and young adults relative to their female counterparts (Coef. = 1.11, *p* = 0.000). Moreover, it was observed that the predictive probability of using mobile phones for STIs education and prevention is high among adolescents and young adults population who owned and used smart phones other than ordinary GSM phones (Coef. = 0.46, *p* = 0.013).

Other explanatory variables such as age, level of education, programme of study, mere ownership of a mobile phone and frequency of mobile phone usage on daily basis were not statistically significant predictors of young adults’ likelihood of using mobile phones for the purposes of education and prevention of STIs. Table 2 shows details of the Univariate Probit regression analysis output.

**Table 2:** Univariate probit regression on predictors of respondents’ likelihood of using mobile phones for STIs education and prevention

	Dependent Variable: use of mobile phone for STIs education and prevention^**^				
Independent variables	Obs.	Coef.	Std. Err	95%[Conf.	Int.]
Age					
18-19 years	29	0.09	0.30	−0.50	0.69
20 years or more	221	Ref.	Ref.	Ref.	
Gender					
Male	149	1.11^†^	0.19	0.74	1.47
Female	101	Ref.	Ref.	Ref.	
^a^Educational level					
Diploma	46	−0.05	0.22	−0.49	0.38
Degree levels (100–400)	204	Ref.	Ref.	Ref.	
Programme of study					
Health sciences	83	−0.16	0.19	−0.53	0.22
Other programmes	167	Ref.	Ref.	Ref.	
Mobile phone ownership					
Yes	230	0.05	0.31	−0.55	0.65
No	20	Ref.	Ref.	Ref.	
Mobile phone type					
Smart phone	144	0.46^†^	0.19	0.10	0.83
GSM phone	106	Ref.	Ref.	Ref.	
Frequency of mobile phone usage					
Every two hours or less	106	−0.28	0.19	−0.65	0.08
Every three hours or more	144	Ref.	Ref.	Ref.	
Probit regression					
Obs	250				
Wald chi2(7)	41.58				
Prov>chi2	0.000				
Pseudo R2	0.16				
Log pseudolikelihood	− 126.20				

Source: Field Data (2016)

Legend: †*p* < .05 (probit regression test, VCE (robust); ^a^Study Level (In Ghana this represents the 4 years of University eduction required to complete a first degree or Bachelor degree; Diploma studies are relatively shorter usually 1 or 2 years ending up in the award of a Diploma, which is lower than a Bachelor Degree); ^**^In this context meant, students who self-reported whether or not they use their mobile phones for the purpose of accessing educational materials on STIs and their prevention. This variable had a binary response outcome “1 = Yes” or “0 = No”. The limitation is that there was no independent verification of the study participants’ responses

## Discussion

Use of mobile phone technology is increasingly becoming an innovative teaching and learning technique in many public and private tertiary institutions in Ghana [24] and other developing countries in Africa [25]. However, the focus has predominantly been on mainstream academic activities such as sharing lecture materials, taking online examinations and student assessment. There is therefore limited empirical evidence on the use of mobile phone technology for public health education endeavours including education on STIs and their prevention.

This study assessed mobile phone use among adolescents and young adults pursuing tertiary education at the University of Ghana, Legon and their perspectives on use of these mobile phones for STIs education and prevention. The higher mobile phone usage (99.2%) among the respondents in this study confirms the World Bank report indicating mobile phone subscription in Ghana has skyrocketed over the years [26].

It was found in this study that ownership of mobile phones by respondents was higher than similar studies in China and Ethiopia with 88.5% and 76.1% ownership rate respectively [27, 28]. These differences might be related to the study setting which in this case consisted of younger University students who have a higher likelihood of using mobile phones and smartphones than the general population. Additionally, the higher ownership of smartphones by respondents in this study demonstrates the increasing popularity of smartphones in sub-Saharan Africa [15]. Furthermore, findings of the current study on onwership of mobile phones could be explained by the fact the study population mainly composed of much younger and educated people than the general Ghanaian population and are therefore more likely to own a smartphone [29]. Also, as the average cost of smartphones declines in LMICs, more people are expected to own these devices.

The high proportion of respondents (70%) who believe in the use of mobile phones for STIs education and prevention suggests the anonymity and confidentiality of mobile phones and their ability to reduce the stigma, discrimination, and shyness associated with young adults’ access to STIs services in health facilities. This response is consistent with similar studies [28, 30, 31] which alluded to similar reasons for preference of mobile phones in accessing STIs information by young adult populations.

Additionally, similar previous studies showed that 95% of respondents in Ethiopia; 68.9% of respondents in China, and 70% respondents in southern California expressed willingness to use mobile phones for STIs care, education and prevention [27, 28, 30]. As discovered in this study, the interest to use mobile phones for STI education and care was equally high among young adults who participated in the study. These findings support the increasing popularity of *mHealth* as an effective innovative method of disseminating sexual and reproductive health information and tips with evidence of improvement in STIs knowledge and testing uptake in Ghana and beyond [32].

The increasing popularity of the use of social networking tools such as Facebook, Twitter, WhatsApp, Blogging and other smartphone applications among adolescents and young adults in this study is consistent with findings in previous publications [33] where these applications have been proven to be effective tools for educating young adults on STIs. These networking tools available on smartphones therefore provide convenient and more cost-effective ways of communication among adolescents and young adults.

It must however be emphasised that even though the use of mobile phones is high, some empirical studies have found knowledge and usage of mobile is not necessarily a pre-requisite for adaption of desirable behaviour in terms of usage for health promotion activities [32, 33]. Nonetheless, the relative better anonymity and privacy afforded by using a mobile phone for sensitive topics like sexual and reproductive health makes it a relatively favorable platform for young adults. It is also important to note that even though mobile phones have been shown to be an acceptable mode of disseminating sexual health information, it might not be effective in all aspects of STIs care such as results notification and post-test counseling [34].

Furthermore, gender is a strong predictor of young adults' likelihood of using of mobile phone mediated means or existing traditional means (i.e. non-ICT or mobile) for self education on STIs and their prevention. As demonstrated in this study, male adolescents and young adults were more likely to use mobile phones for the purposes of STIs education and prevention relative to their female counterparts. Perhaps the females unlike males are more inclined to shy away from STIs discussions via this platform due to its sensitive nature. This observation thus call for a complementary youth friendly approach to STIs education by blending existing traditional approaches with *mHealth* innovations. For instance, the human touch afforded by the face-to-face method of communication is usually absent in digital means of communication. Therefore, mobile phone-based programmes for STIs education should not be a replacement of the existing traditional STIs education media but a complementary approach.

It was found in this study that gender, type of phone and brand of the mobile phone were strong predictors of adolescents and young adults’ use of mobile phones for STIs education and prevention. The study found that even though females were generally more interested in health and health information than males, the probability of they using mobile phone for STIs prevention was less than males. Some empirical studies have found that females are more likely to enage in health promotion behaviours more than men [35, 36], but these previous empirical observations contrast with findings of this study in terms of the use of mobile phone for health promotion activities by females regarding STIs.

Gender differences are pronounced in sexual and reproductive health, and this is being reinforced by the vulnerabilities of women to STIs and the socio-cultural norms in Ghana. In some instances, men easily take decisions on testing voluntarily for STIs, as compared to most women who have to get tested as part of health care services received [37]. For instance, while Prevention of Mother to Child Transmission (PMTCT) of STIs are available to capture women with STIs, there is no corresponding health system arrangement that deliberately captures their male counterparts [37, 38].

The study also made significant observations on the penetration of smartphones in the Ghanaian market. Respondents who use smartphones were more positive that adolescents and young adults would be interested in a mobile phone-based programme for STIs education than ordinary phone users. Thus, possession of a smartphone constituted a significant predictor of the use of mobile phone for STIs education and prevention among university students who participated in this study. Smartphones act as miniature computers with a variety of features that allow for internet, mobile applications, video conferencing, and Global Positioning System (GPS) navigation.

In contrast, only text messaging, which is the main functionality of a mobile phone is increasingly becoming unpopular among young adults hence the increasing adoption of smartphones to access applications such as Twitter, Facebook, WhatsApp, Messenger, and Instagram. The occasional use of text messaging among the respondents relative to their frequent use of mobile applications and the mobile web could be attributed to the increased adoption of smartphones in Ghana.

Also, the 160-character limit for Short Message Service (SMS), and cost per text message which constitute the main communication medium for feature phones as against the flexibility of using smartphones applications makes feature phones obsolete and deemed archaic among adolescents and young adults. In light of these revelations, it would be more acceptable by the youth if future mobile phone-based programmes for STIs education and prevention solely utilize smartphones for such interventions.

## Conclusion

A large proportion of adolescents and young adults reported having a mobile phone. The findings show that the willingness and interest to use mobile phones for STIs education and prevention is high. Also, gender and type of mobile phone used were significant predictors of young adults’ probability of using mobile phone for STIs education and prevention, perhaps due to due flexibility of use.

Given the stigma, discrimination, shyness, and fear associated with adolescents and young adults’ access to STIs services from hospitals, these findings have important public health implications in the fight against STIs among these age groups as emerging mobile technologies seek to eliminate these barriers to STIs services. A mobile phone programme for STIs education that uses mobile application appears feasible, acceptable sustainable over time for adolescents and young adults. This feasibility presupposes that future mobile phone programmes for STIs education could utilise innovative mobile applications on smartphones for young adults who are often the most vulnerable group for contraction and transmission of STIs. Nonetheless, the limitations associated with the use of mobile phone technology for health promotion activities are acknowledged and should be taken into consideration by policy makers and health managers in the area of sexual and reproductive health in Ghana.

### Limitations

The researchers acknowledge some limitations associated with the study. First, the study was conducted in only one out of over ten (10) public universities in Ghana. The study scope thus, poses generalizability challenges to other parts of the country since the youth dynamics and use of mobile phones significantly vary in the different regions of Ghana. Moreover, the use of convenience sampling in recruiting the respondents poses potential self-selection bias where adolescents and young adults who accepted to participate in the study might have been naturally interested and perhaps more enthusiastic in mobile phone usage.

Finally, the responses of participants were self-reported which could have introduced socially desirable responses since they were not independently verified by the researchers. Future researchers should consider complementing this study design with independent means of verifying the participants’ responses.

Notwithstanding the above limitations, the findings remain relevant because mobile phone penetration in Ghana is monumentally high but leveraging this success for education of the teaming youth on STIs is yet to be fully realized. Furthermore, the topic is relevant because STIs including HIV/AIDs are the leading of causes of morbidity and mortality among the youth in Africa including Ghana. Thus, innovative approaches such mobile phone technology for health education and prevention of STIs is timely, albeit the study limitations.

### Implications for public health policy and interventions design

Based on the empirical findings from this study, the following recommendations are proposed to guide the development and implementation of public health interventions geared towards STIs education and prevention among the youth in Ghana and Africa as a whole.

1. Public health education on STIs should incorporate statutory eHealth and mHealth components to promote acceptability among the youth
2. Interventions towards STIs control and prevention among the youth should emphasise gender roles and dynamics particularly with respect to role of females who are most vulnerable yet less receptive to mobile-based STIs innovations
3. There is the need to step-up existing efforts on men inclusion in women’s health, as advocated in Prevention of Mother to Child Transmission (PMTCT), to help support females and also better inform men on misconceptions on STIs.
4. Design and implementation of STIs control and prevention interventions should actively involve relevant stakeholders in the Information Communication Technology (ICT) industry to design youth friendly mobile applications on STIs education and prevention in LMICs such as Ghana

## Additional file

Additional file 1: Questionnaire. (PDF 47 kb)

## Data Availability

The datasets used and/or analysed in this study are available from the corresponding author on reasonable request.

## Abbreviations

AIDS: Acquired Immune Deficiency Syndrome
GPS: Global Positioning System
HIV: Human Immune Virus
LMICs: Lower- and Middle-Income Countries
*mHealth*: Mobile-Health
PMTCT: Prevention of Mother to Child Transmission
SMS: Short Message Service
STIs: Sexually Transmitted Infections
