# Supplementary material for: Determinants of use of mobile phones for sexually transmitted infections (STIs) education and prevention among adolescents and young adult population in Ghana: implications of public health policy and interventions design
Source: Reprod Health. 2019 Aug 9;16:120. doi: 10.1186/s12978-019-0763-0 (PMC6688338; doi:10.1186/s12978-019-0763-0)
Supplement: Supplementary file 1 — Questionnaire. (PDF 47 kb) [file 12978_2019_763_MOESM1_ESM.pdf]

UNIVERSITY OF GHANA, LEGON
COLLEGE OF HEALTH SCIENCES
SCHOOL OF NURSING

STRUCTURED QUESTIONNAIRE

Preamble and Informed Consent

I Abdulai Abdul Fatawu is a graduate from the School of Nursing, University of Ghana Legon. I am collaborating with other investigators from the University of Health and Allied Sciences, Ho Ghana to explore the use of cell phones for STI/HIV education and prevention among university students between the ages of 18-24. This study is important because there is no research in Ghana that seeks to establish the feasibility and acceptability of cell-phone based programs among young people to inform the development of tailored programme. Attached is a questionnaire about your demographic information, use of mobile phones, STI/HIV health seeking practices and the likelihood to use mobile phones for HIV/AIDS prevention and education. You should understand that your participation is entirely voluntary and you are not required to disclose your identity on the questionnaire. Data from this study will be handled in a private and confidential manner. This questionnaire will take approximately 7-10 minutes to complete.

Do you consent to voluntarily participate in this study after satisfactorily understanding the above explanation?

If yes please Sign or Thumb-print Here____

For further enquiries on this study please contact Abdulai Abdul Fatawu via email address: abdulaiabdulfatawu@yahoo.com

INSTRUCTIONS: Please tick in the boxes provided after each option as your most appropriate response

SECTION A. DEMOGRAPHIC INFORMATION

1. What is your age?
   A. 18-19 [ ]1
   B. 20-21 [ ]2
   C. 22-23 [ ]3
   D. 24 [ ]4

2. What is your gender?
   A. Male [ ]1
   B. Female [ ]2

3. What is your level of studies?
   A. Diploma [ ]1
   B. Level 100 [ ]2
   C. Level 200 [ ]3
   D. Level 300 [ ]4
   E. Level 400 [ ]5

4. What is your study department?
   A. Business administration [ ]1
   B. Health Sciences [ ]2
   C. Arts [ ]3
   D. Science and Technology [ ]4
   E. Others [ ]5

5. What is your ethnic affiliation?
   A. Akan [ ]1
   B. Ga-Adangbe [ ]2
   C. Ewe [ ]3
   D. Dagomba [ ]4
   E. Frafra [ ]5
   F. Others (please specify)………………………………………………

6. What is your hall of residence? (Please specify)………………….………..

SECTION B. USE OF CELL PHONE TECHNOLOGY

7. Do you own a mobile phone?
   A. Yes [ ]1
   B. No [ ]2

8. If yes to question 7, what type of phone is it?
   A. Smartphone [ ]1
   B. An ordinary GSM phone [ ]2

9. What brand of phone do you use?
   A. Nokia [ ]1
   B. Windows Mobile [ ]2
   C. Android [ ]3
   D. Iphone [ ]4
   E. Blackberry [ ]5
   F. Others (Please specify)………………………………………

10. How much time do you use your cell phone daily?
   A. Every 2 hours and below [ ]1
   B. Every 3-4 hours [ ]2
   C. Every 5-6 hours [ ]3
   D. 7-8 hours [ ]4
   E. 9 hours and above [ ]5

Instruction: From a scale of 1 to 5 where 1=very frequently, 2=frequently, 3=occasionally, 4=rarely 5= never, please indicate your frequency of use with any of the following cell phone technologies

	VF	F	O	R	N
11. Text messaging	1	2	3	4	5
12. Mobile apps	1	2	3	4	5
13. Phone call	1	2	3	4	5
14. Mobile web	1	2	3	4	5

Legend: 1=VF (very frequently), 2= F (frequently), 3= O (occasionally), 4= R (rarely) 5= N (never)

Instruction: From the scale of 1 to 5, how would you rate the ease of use with the following cell phone technologies?

	EE	VE	ME	SE	NE
15. Text messaging	1	2	3	4	5
16. Mobile apps	1	2	3	4	5
17. Phone call	1	2	3	4	5
18. Mobile web	1	2	3	4	5

Legend: 1=EE (Extremely easy), 2= VE (Very easy), 3= ME (Moderately easy), 4= SE (Slightly easy) 5= NE (Not easy)

SECTION C. YOUNG ADULTS USE OF CELL PHONES FOR STI/HIV PREVENTION AND EDUCATION

19. Which of the following do you think prevents adolescents and young people from accessing STI/HIV services from hospitals?
   A. Shyness [ ]1
   B. Fear [ ]2
   C. Distance [ ]3
   D. Unknown Locations [ ]4
   E. Cost [ ]5
   F. They don’t need it [ ]6

G. Others (please specify)………………………………..

20. Do you think cell phones can be used in STIs prevention and eliminate of barriers in question 19?
   A. Yes [ ]1
   B. No [ ]2
   C. Not sure [ ]3

21. Which of these do you think would be the appropriate means for STI/HIV education and prevention for young adults?
   A. Text messaging [ ]1
   B. Use of mobile apps [ ]2
   C. Phone calls [ ]3
   D. Mobile webs [ ]4
   E. Others (please specify)…………………………………

22. Do you think young people will be interested in a cell phone program that allows them to have a personal consultation with a doctor or nurse concerning STI/HIV?
   A. Very interested [ ]1
   B. Somehow interested [ ]2
   C. Not interested [ ]3

THANK YOU FOR YOUR TIME
